# Isolation of starch and protein degrading strain *Bacillus subtilis* FYZ1-3 from tobacco waste and genomic analysis of its tolerance to nicotine and inhibition of fungal growth

**DOI:** 10.3389/fmicb.2023.1260149

**Published:** 2023-11-16

**Authors:** Changwen Ye, Dandan Liu, Kuo Huang, Dong Li, Xinxin Ma, Yiying Jin, Hanguo Xiong

**Affiliations:** ^1^College of Food Science and Technology, Huazhong Agricultural University, Wuhan, China; ^2^China Tobacco Standardization Research Center, Zhengzhou Tobacco Research Institute of CNTC, Zhengzhou, China; ^3^School of Environment, Tsinghua University, Beijing, China

**Keywords:** tobacco waste, *Bacillus subtilis*, whole genome sequencing, bacteriostasis, composting, nicotine

## Abstract

Aerobic fermentation is an effective technique for the large-scale processing of tobacco waste. However, the specificity of the structure and composition of tobacco-derived organic matter and the toxic alkaloids in the material make it currently difficult to directly use microbial agents. In this study, a functional strain FYZ1-3 was isolated and screened from thermophilic phase samples of tobacco waste composting. This strain could withstand temperatures as high as 80°C and grow normally at 0.6% nicotine content. Furthermore, it had a strong decomposition capacity of tobacco-derived starch and protein, with amylase activity of 122.3  U/mL and protease activity and 52.3  U/mL, respectively. To further understand the mechanism of the metabolic transformation of the target, whole genome sequencing was used and the secondary metabolite gene cluster was predicted. The inhibitory effect of the strain on common tobacco fungi was verified using the plate confrontation and agar column methods. The results showed that the strain FYZ1-3 was *Bacillus subtilis*, with a genome size of 4.17  Mb and GC content of 43.68%; 4,338 coding genes were predicted. The genome was annotated and analyzed using multiple databases to determine its ability to efficiently degrade starch proteins at the molecular level. Moreover, 14 functional genes related to nicotine metabolism were identified, primarily located on the distinct genomic island of FYZ1-3, giving a speculation for its nicotine tolerance capability on the molecular mechanism. By mining the secondary metabolite gene cluster prediction, we found potential synthetic bacteriocin, antimicrobial peptide, and other gene clusters on its chromosome, which may have certain antibacterial properties. Further experiments confirmed that the FYZ1-3 strain was a potent growth inhibitor of *Penicillium chrysogenum*, *Aspergillus sydowii*, *A. fumigatus*, and *Talaromyces funiculosus*. The creation and industrial use of the functional strains obtained in this study provide a theoretical basis for its industrial use, where it would be of great significance to improve the utilization rate of tobacco waste.

## 1 Introduction

China is the largest producer and consumer of tobacco in the world (Zhang G. et al., 2022). The amount of tobacco waste generated in 2020 has exceeded 2.6 million tons (Zhang Y. et al., 2022). Tobacco waste is a valuable biomass resource (Wang J. et al., 2022), while traditional treatment means often involve random piling or centralized incineration, which not only leads to serious secondary pollution but also to resource waste (Dobaradaran et al., 2020; Koutela et al., 2020; Lee et al., 2021). Studies have confirmed that the aerobic fermentation of tobacco waste is an effective method for the large-scale utilization of waste resources, and exogenous microbial agents are usually added for high-temperature aerobic fermentation to fasten the process of composting and production of organic fertilizer that has added value (Kayikçioglu and Okur, 2011; Awasthi et al., 2017). Among them, *Bacillus subtilis* has become the most widely used microbial strain owing to its rapid growth rate, high tolerance, and strong degradation ability (Nayak, 2020). The screening and identification of *Bacillus* spp. to make complex microbial agents can significantly shorten the composting cycle and improve the degradation rate of sludge, garden waste, food waste, etc. (Fang et al., 2019; Liu et al., 2022a,b; Wang M. et al., 2022).

However, tobacco waste usually contains toxic alkaloids, among which, nicotine accounts for >95% (Zhou, 2008). The U.S. Environmental Protection Agency had added nicotine to the list of environmentally restricted release inventories in 1994, and the European Union also introduced laws stipulating that tobacco waste with a mass fraction exceeding 0.05% should be classified as hazardous waste (Ashengroph, 2015; Weishaar et al., 2016). Tobacco waste usually contains 0.1%–4.0% (mass fraction) of nicotine (Civilini et al., 1997), and some studies have shown that nicotine has a certain inhibitory effect on microbial metabolic pathways, gene expression, cell reproduction, enzyme activity, and other biological functions (Yildiz, 2004; Fitzpatrick, 2018; Nölke et al., 2021). Additionally, there are significant differences in the structure and background impurities between tobacco-derived starch and proteins and ordinary starch and proteins according to current studies (Masson and Rossignol, 1995; Song et al., 2010; Wang et al., 2012). Although there are some studies and applications on efficient organic matter-degrading bacterial agents, there are no relevant reports on bacterial agents that can degrade tobacco-derived organics and withstand nicotine at high temperatures. Therefore, it is necessary to identify a functional strain that can simultaneously withstand high temperatures and nicotine content, as well as have a high degradation ability of tobacco source organic matter.

In this study, tobacco waste was selected as the sampling object, and tobacco starch and tobacco proteins were used as the sole carbon sources as the enrichment medium to isolate the strains that could degrade tobacco-derived organic matter at 80°C. The strain with the maximum amylase and protease activities was selected as the functional strain. This study utilized tobacco waste composting samples during the high-temperature period to screen bacteria, using tobacco starch and tobacco protein as the only carbon sources. Enrichment took place at 80°C, followed by preliminary screening using a transparent hydrolysis ring and enzyme activity determination to obtain a strain capable of withstanding high temperatures, degrading tobacco organic matter, and displaying nicotine tolerance. The functional strain with the greatest comprehensive amylase and protease activity was chosen as the target for further analysis. Moreover, the tolerance of functional strains to nicotine levels was further determined. The classification status of functional strain was determined using phylogenetic analysis based on 16S rRNA gene sequencing and average nucleotide identity (ANI) analysis.

Studying the basic genomic information of functional strains can help researchers further understand the mechanism of metabolic transformation of functional strains toward target substances, and also facilitate the in-depth understanding of the relationship between genes and proteins, metabolic function, and individual behavior. Therefore, the whole genome sequence of the functional strain was determined to determine its ability to efficiently degrade starch and protein at the molecular level, and to further explore whether there is a secondary metabolite gene cluster on its chromosome that synthesizes bacteriocins and antimicrobial peptides. The growth-inhibition ability of common tobacco fungi was verified using the confrontation plate culture and agar column methods.

The thermophilic strain obtained in this study was resistant to nicotine and had high degradation ability to tobacco source organics. Using whole genome sequencing, it was found that it also has a certain antibacterial effect, producing organic fertilizers capable of biological control, which providing a theoretical basis for the creation and industrial application of functional microbials in the later stage. Thus, this study could be of significance for improving the utilization rate of tobacco waste resources.

## 2 Materials and methods

### 2.1 Materials

#### 2.1.1 Source of samples

Compost samples were obtained from tobacco waste piles in their natural state from Luliang County, Qujing City, Yunnan Province of China.

#### 2.1.2 Culture media

Bacterial culture medium: LB medium (tryptone 10 g/L, yeast extract g/L, NaCl 10 g/L, pH 7.2–7.4); fungal medium: potato dextrose agar (PDA) medium (potato 200 g/L, glucose 20 g/L, natural pH). Agar powder (20 g/L) was added to the solid medium and sterilized at 121°C for 20 min.

Tobacco starch-screening medium: tobacco starch 1 g/L, NaCl 5 g/L, yeast powder 2 g/L, agar powder 15 g/L. Tobacco-derived protein-screening medium: tobacco protein 2 g/L, sodium chloride 5 g/L, agar powder 15 g/L.

Nicotine-fermentation medium: 100% nicotine was added to LB liquid medium according to the volume ratio to prepare nicotine media (0.3, 0.6, 0.9, 1.2, and 1.5%).

### 2.2 Methods

#### 2.2.1 Isolation and screening of the functional strain

Sampling and enrichment: 5 g of the pile was weighed using the cross-sampling method. Next, 45 mL of sterilized phosphate buffer solution was added and the sample was homogenized for 10 min. The mixture was heated at 80°C in a water bath for 15 min. Then the heated liquid was centrifuged at 3000 rpm/min for 10 min and 1 mL of the supernatant was added to a liquid medium containing 50 mL of tobacco starch and that of tobacco protein, respectively. The two media were incubated at 37°C and 180 r/min for 48 h.

Screening of bacterial strains: after heating the above bacterial solution in a water bath at 80°C for 15 min, gradient dilution was performed. Next, 100 μL of bacterial suspensions with five dilutions of 10^−4^ to 10^−8^ were drawn and inoculated on tobacco starch or tobacco protein screening medium and cultured at 37°C for 48 h in a constant temperature and humidity box. The tobacco starch culture medium was dripped with iodine solution, and single-colony strains with larger transparent circles were visually selected. The diameter of the transparent circles and colony diameters were measured using a digital vernier caliper and the ratio of the two (HC value) was calculated.

Determination of enzyme activity: using the iodine colorimetric method, the amylase secreted by the bacterial strain interacts with starch under certain conditions. The reaction solution is detected by a spectrophotometer at a specific time to color with iodine, and the results are obtained by referring to the alpha-amylase activity table. Specific operating procedures were based on China National Standard (GB1886.174-2016, 2016). Protease activity is often measured using casein as a substrate to produce amino acids under specific hydrolysis conditions. Its activity is measured by the amount of tyrosine produced, which is evaluated by the Folin-phenol reagent method. Specific operating procedures were based on China National Standard (GB/T 23527-2009, 2009).

Determination of nicotine tolerance: 100 μL of strains in the logarithmic phase were added to different concentrations of the nicotine fermentation medium and cultured in a shaker at 37°C and 220 rpm for 16 h, followed by measurement of the OD_600_. An equal volume of sterile water was used as the blank control group.

#### 2.2.2 Functional strain identification and whole genome sequencing

The genomic DNA of FYZ1-3 was extracted using the FastPure Gel DNA Extraction Mini Kit (Vazyme, Nanjing, China) according to the manufacturer’s instructions. The pair of bacterial universal primer (upstream primer 27F: 5′-AGAGTTTGATCCTGGCTCAG-3′; downstream primer 1492R: 5′-GGYCCCTTGTTACGATT-3′) was used as the 16S rDNA amplification primer. Polymerase chain reaction (PCR) amplification reaction system (50 μL): 2 μL upstream and downstream primers (10 pmol/μL), 2 μL 10× PCR buffer (containing Mg^2+^), 0.6 μL Taq DNA polymerase, 4 μL dNTP mix. PCR amplification reaction conditions: 94°C, pre-denaturation for 5 min; denaturation at 94°C for 30 s, annealing at 55°C for 30 s (renaturation), extension at 72°C for 1 min, 30 cycles; 72°C for 10 min. PCR products were sent to Shenzhen Huada Gene Co., Ltd. for sequencing and the results were compared with the sequence in the NCBI database for BLAST homology.

Genomic DNA extraction and database construction: the genomic DNA of the screened functional strain, FYZ1-3, was extracted using the STE method, which was comprised of a mixture of sodium chloride-Tris-HCl-EDTA to create a protective environment for the DNA (Pan et al., 2006; Shui et al., 2014). In addition, the purity and integrity of the DNA were evaluated through agarose gel electrophoresis and quantified utilizing Qubit. The SMRT Bell library was constructed using the SMRT Bell^™^ template kit. The constructed library was quantified by Qubit concentration, and the size of the inserted fragment was detected using Agilent 2100. The PacBio platform was utilized for sequencing purposes whereby the PacBio RS II featured single molecule real-time sequencing technology. Upon the complex creation with DNA and polymerase and its containment within the ZMW (zero mode waveguide), four diverse fluorescent labeled dNTPs entered the detection area randomly through Brownian motion and bind with the polymerase. Chemical bonds required more time to form with the template-matching base in comparison to the other bases. The duration of fluorescence information’s existence could discern between matched and free bases. Analyzing the relationship between time and four fluorescence information types enabled sequencing of DNA template sequences.

#### 2.2.3 Validation of the fungal-inhibition ability of the functional strain

Confrontation plate culture method: four typical tobacco pathogens, *Penicillium chrysogenum*, *Aspergillus sydowii*, *A. fumigatus* and *Talaromyces funiculosus*, were selected as indicators. The active bacterial solution was coated on PDA plates and incubated at 28°C for 72 h. After a single colony was established, the suitable bacteria were selected by means of a sterilized puncher and shaped into a 5 mm bacterial cake. The cake was first connected to a fresh PDA plate and the functional strain was spotted at a distance of 2.5 cm from the cake; a double-distilled water (ddH_2_O)-spotted plate was used as a control. Fungi were cultured in a constant-temperature incubator at 28°C for 72 h, and fungal growth was recorded.

Agar column method: the fermentation supernatant of the functional strain was added to the PDA medium in a 1:10 (v/v) ratio, and the activated indicator bacterial cake was punched with a 5 mm sterilization punch and placed in the center of the PDA medium. A plate with the same amount of ddH_2_O was used as a control. The cells were cultured in a constant temperature incubator at 28°C for 48 h. The cross-crossing method was used to estimate the scattered area of bacteria and spores. The antibacterial rate was calculated as follows:


(1) $$\text{Inhibition rate}\;\left(\%\right)=\frac{\begin{array}{l} \text{Bacterial cake area in the blank group}-\\ \text{Bacterial cake area in the experimental group} \end{array}}{\text{Bacterial cake area in the blank group}}\times 100$$

#### 2.2.4 Functional annotation of genes

The default algorithm of GeneMarkS (Version 4.17) was used to predict the encoding genes of the sequenced genome. Based on sequence composition, the IslandPath-DIOMB software (Version 0.2) was used to predict gene islands, which determine gene islands and potential horizontal gene transfer by detecting nucleotide bias and mobility genes (such as transposases or integrases) in the sequence. With a library based on the specified type of HMM algorithm built, the antiSMASH 4.0.2 program was used to predict and identify all known secondary metabolic clusters in the genome. The annotated universal functional databases used in this study include GO, KEGG, and CAZy. The basic steps of functional annotation are as follows: (1) perform diamond alignment of the predicted gene protein sequence with various functional databases(evaluation ≤1e^−5^); (2) filter alignment results: for each sequence’s alignment results, select the alignment result with the highest score (default identity ≥40%, coverage ≥40%) for annotation. Lastly, whole genome sequencing data of the measured functional strains were submitted to the NCBI database, and the GenBank accession number is SRR24954540.

## 3 Results

### 3.1 Screening and characterization of functional strain FYZ1-3

**Figure 1:**
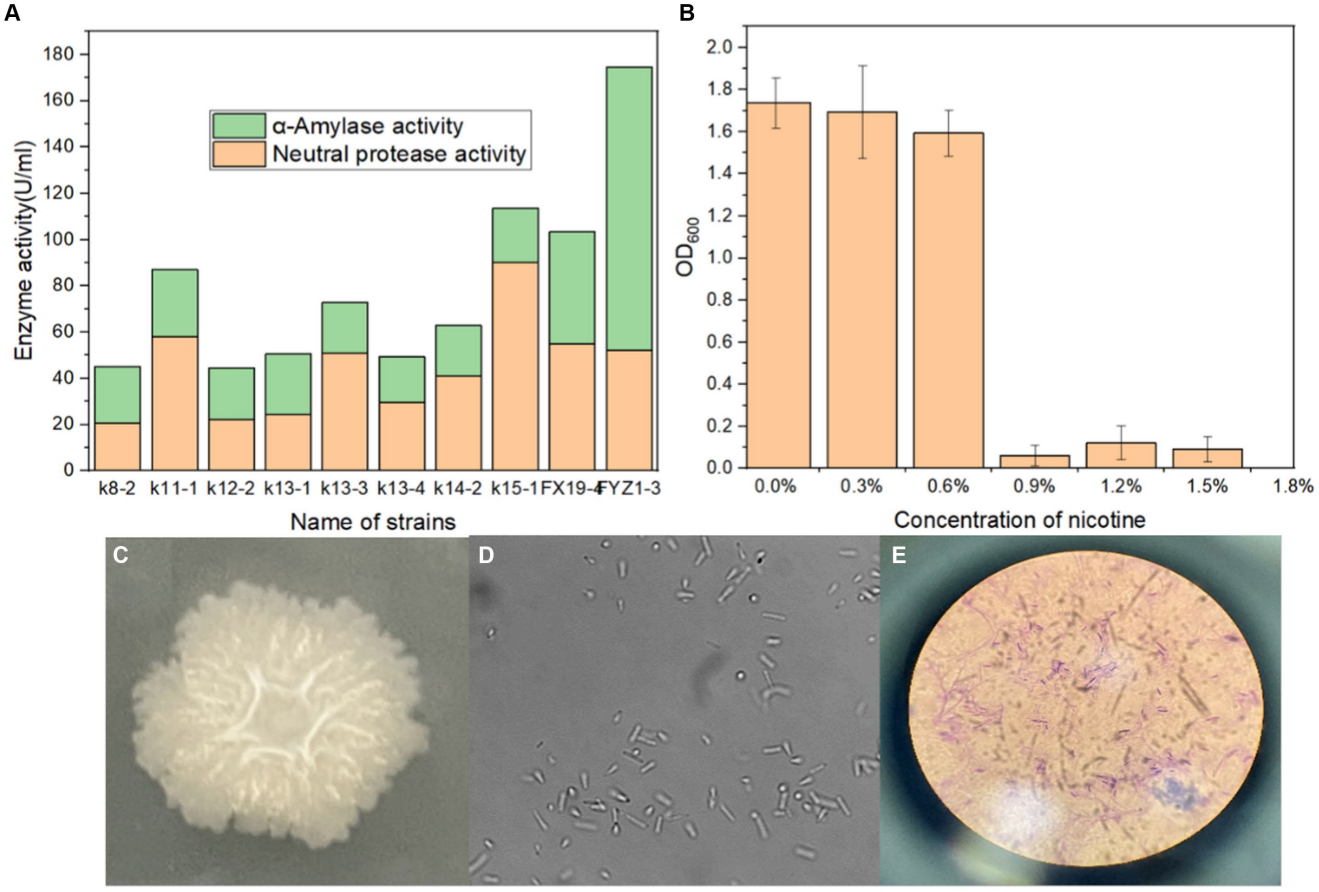
Screening process and characteristics of the FYZ1-3 strain: **(A)** starch and protein enzyme activity; **(B)** nicotine tolerance; **(C)** colony morphology; **(D)** microscope images; **(E)** Gram staining.

A total of 31 culturable single colonies were obtained through enrichment culture of tobacco waste compost samples at high-temperature period. Next, 10 strains with clear degradation abilities on starch and protein medium were then selected based on the hydrolysis transparent circle diameter to colony diameter ratio (H/C) on selective medium, as shown in Table 1. Finally, the amylase activity and protease activity of these 10 strains were determined, and the rescreening results were shown in Figure 1A. It could be seen that FYZ1-3 exhibit the highest amylase activity of 122.3 U/mL among all ten strains, which was considerably higher than that of the other strains (20.0–48.4 U/mL). The protease activity of 10 bacterial strains ranged from 20.7 to 90.2 U/mL. Despite not displaying the highest protease activity, strain FYZ1-3 demonstrated a value of 52.3 U/mL, which was not significantly different from the highest recorded value. Furthermore, its overall enzyme activity was the highest among all strains evaluated. Therefore, FYZ1-3 was chosen as the functional strain for the subsequent experiments. The FYZ1-3 strain was inoculated in a nicotine fermentation medium with varying concentration gradients, and the OD_600_ was measured after 16 h. The results indicated that the FYZ1-3 strain could tolerate a nicotine concentration of 0.6%, as shown in Figure 1B. Compared with the same type of high temperature and nicotine resistant *B. subtilis* B5221 (Wang et al., 2012), the amylase activity of FYZ1-3 was 122.3 U/mL, which was much higher than the highest enzyme activity of 39.9 U/mL after optimization of culture conditions. Although the protease activity of FYZ1-3 was only 52.3 U/mL, it still exceeded that of the H010 strain screened by Li et al. (2012), which measured 39.2 U/mL. The FYZ1-3 strain was demonstrated extensive functionality, thriving in an environment with a temperature of 80°C and a nicotine content of 0.6%. This strain also exhibited a superior capacity to degrade tobacco starch and protein. As shown in Figures 1C–E, the analysis of single bacterial characteristics, including colony morphology, microscopic cell structure, and Gram staining, identified strain FYZ1-3 as *Bacillus* sp. The colony appeared opaque and milky white with visible surface folds. Rod-shaped cells with visible spore structures were observed under the microscope, and Gram staining was positive.

**Table 1:** Results of initial screening of strains from selected medium.

Item		k8-1	k11-1	k12-2	k13-1	k13-3	k13-4	k14-2	k15-1	FX19-4	FYZ1-3
Starch selective medium	H/C	2.82	2.22	2.57	2.48	2.31	3.09	2.51	2.24	2.06	2.74
	Image	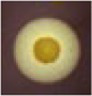	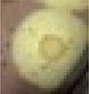	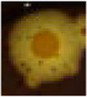	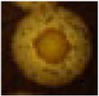	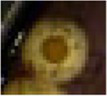	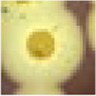	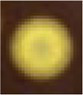	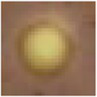	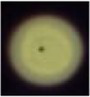	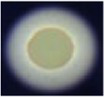
Protein selective medium	H/C	1.43	1.17	1.48	1.32	1.15	1.30	1.20	1.17	3.40	1.15
	Image	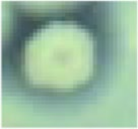	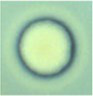	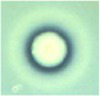	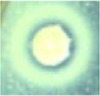	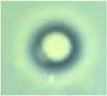	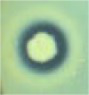	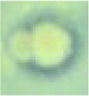	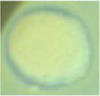	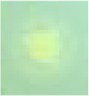	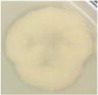

### 3.2 Molecular biology-based identification and whole genome sequencing of functional strains

#### 3.2.1 Phylogenetic tree and ANI analysis based On 16S rRNA gene sequencing

**Figure 2:**
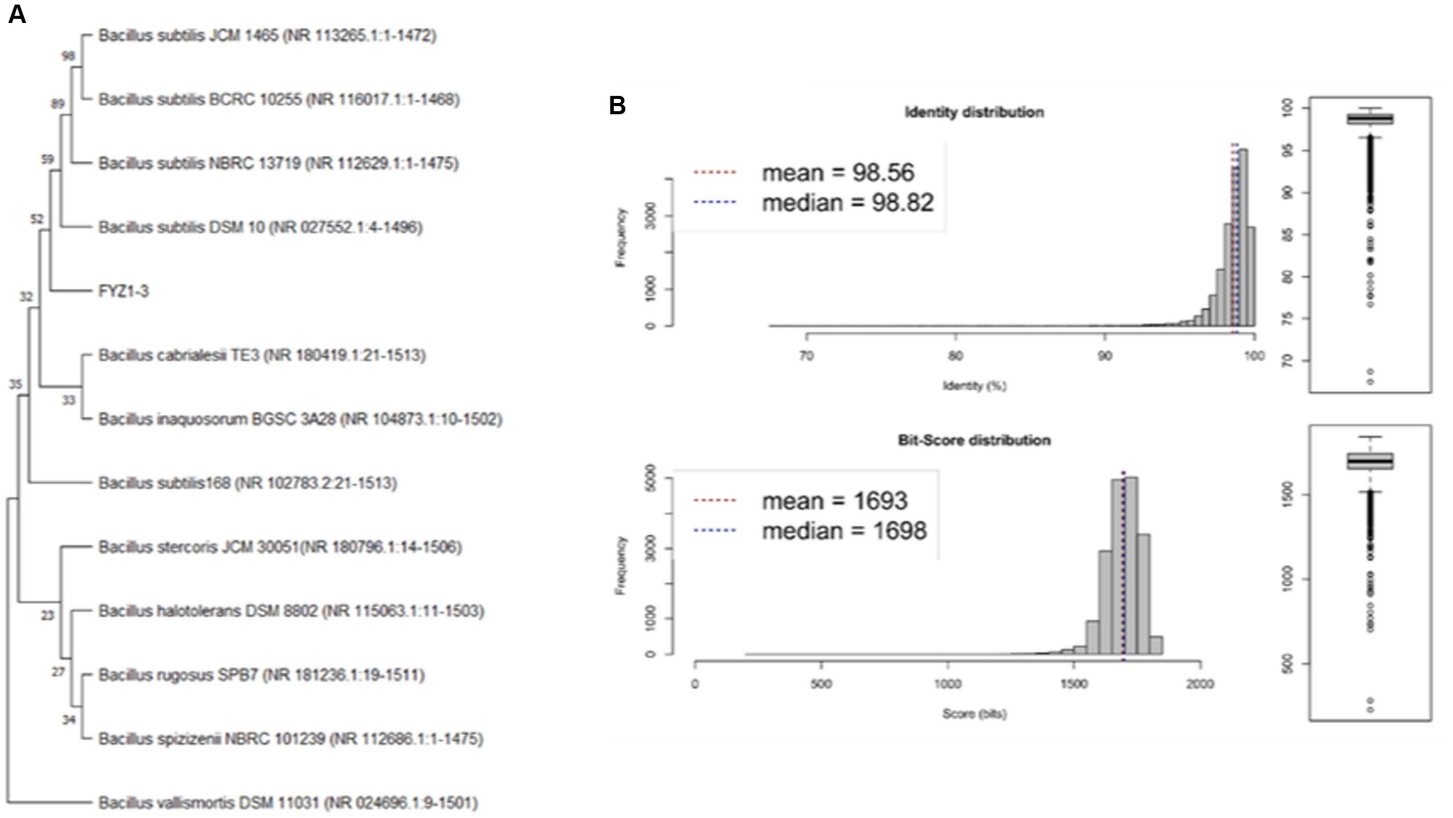
Analysis and identification of strain FYZ1-3: **(A)** 16S rRNA phylogenetic tree; **(B)** ANI value analysis diagram of DSM 10 with the closest genetic relationship.

16S rRNA gene sequencing results of the selected strain FYZ1-3 were uploaded to the NCBI database for BLAST comparison. The similarity of the FYZ1-3 strain with the model strains *B. subtilis* DSM 10, *B. subtilis* NBRC 13719, and *B. subtilis* BCRC 10255 was 100%. The phylogenetic tree was constructed using the neighbor-joining method and MEGA software (version 11) as shown in Figure 2A. *B. subtilis* FYZ1-3 was clustered with other *B. subtilis* spp. and had the closest relationship with *B. subtilis* DSM 10 strain. Among them, based on the information in the GeneBank database, *B. subtilis* DSM 10 strain (GeneBank No.: CP120681.1) was isolated from fermentation materials in western Africa. This high-temperature fermentation environment is similar to the high-temperature fermentation environment of tobacco waste, which may be a reason why DSM 10 and FYZ1-3 strains are closely related. *B. subtilis* DSM10 has been used as a model strain and is widely used as a reference strain in various studies (Lilge et al., 2021).

ANI is mainly used to evaluate the genetic relationship between species at the genome-wide level (Arahal, 2014). Based on the results from 16S rRNA gene sequencing, *B. subtilis* DSM 10 having the highest similarity with the 16S rRNA gene sequence of FYZ1-3 was selected. The complete genome sequence was downloaded from the Assembly database of NCBI, and the ANI value between the FYZ1-3 and *B. subtilis* DSM 10 strains was calculated using JSpecies. Figure 2B shows the homology between FYZ1-3 and *B. subtilis* DSM 10 to be 98.56%, which is higher than the classification threshold of 95%. It was determined that FYZ1-3 and *B. subtilis* DSM 10 were the same species; therefore, it was named *B. subtilis* FYZ1-3.

#### 3.2.2 Genome overview and gene island prediction of the FYZ1-3 strain

**Figure 3:**
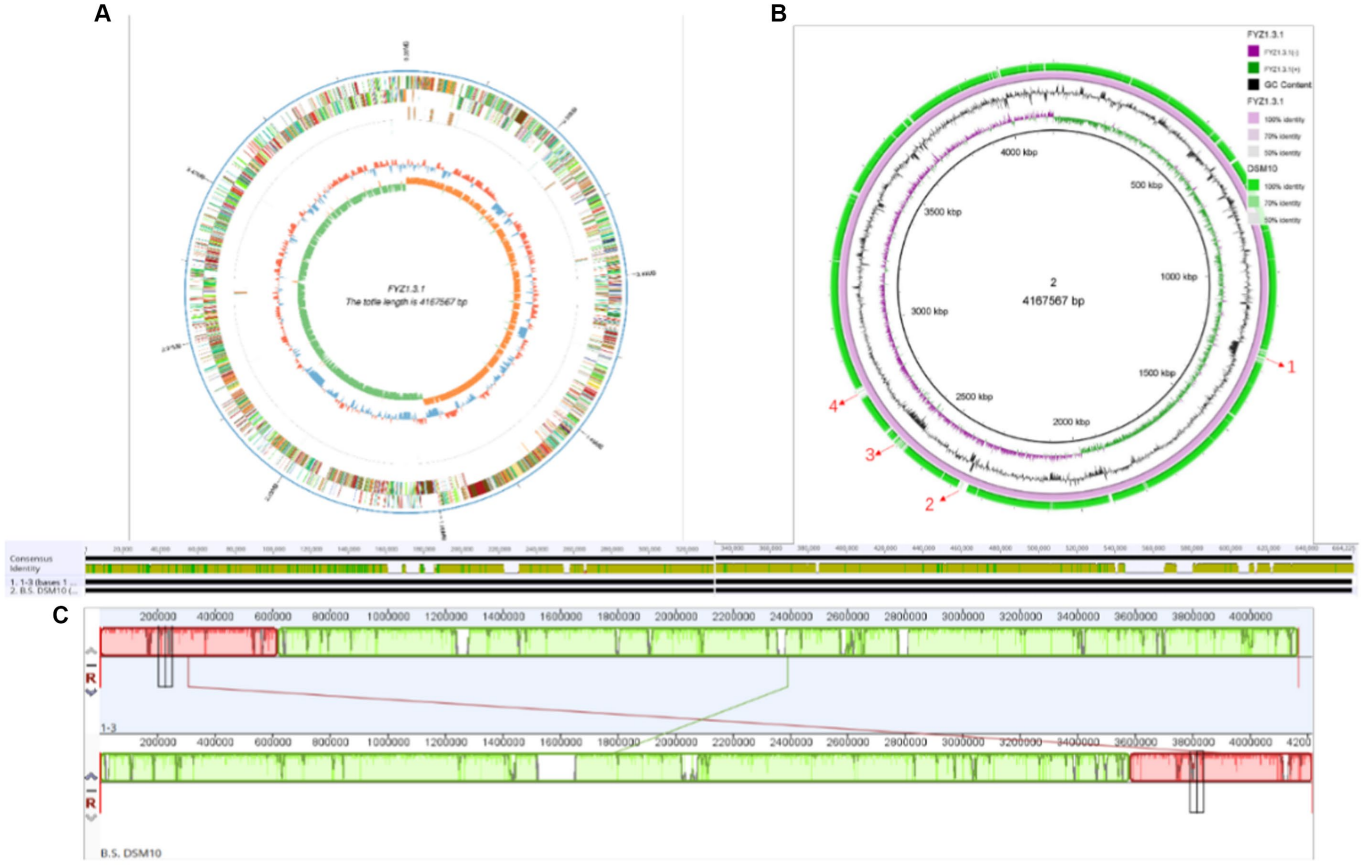
Genome analysis of the strain FYZ1-3: **(A)** whole genome map of strain FYZ1-3; **(B)** comparison of genomic differences between the FYZ1-3 and *B. subtilis* DSM 10 strains; **(C)** analysis of genome collinearity between the FYZ1-3 and *B. subtilis* DSM 10 strains.

PacBio Sequel is a third-generation sequencing platform based on nanopore single-molecule real-time technology, which has the advantages of long sequencing reads, high-throughput analysis, and high accuracy (Mclaughlin et al., 2021). In this study, the whole genome of strain FYZ1-3 was sequenced using the PacBio Sequel sequencing platform, and the genome assembly of the sequence was performed using SMRT Link assembly software to obtain 1 contig. After the data were corrected by Illumina Nova Seq PE150 sequencing results, the chromosome sequence of strain FYZ1-3 was assembled into a circular genome. The whole genome spectrum is shown in Figure 3A. The genome size of the FYZ1-3 strain was 4,167,567 bp, the average GC content was 43.68%, and there were no plasmids. In addition, the genome also contains 86 tRNA genes and 30 rRNA genes.

Genomic island is a gene cluster that can be horizontally transferred in the microbial genome. The transfer of genomic islands is a way of microbial gene exchange, which can improve the diversity of microorganisms and their adaptability to the environment. It is of great significance in the evolutionary analysis of bacteria and the study of special functions that may be obtained during evolution (Dhillon et al., 2015). As shown in Table 2, 15 genomic islands were predicted on the chromosome of strain FYZ1-3. Among the 15 genomic islands, GIs011 was the longest genomic island with a length of 47,019 bp. GIs013 was the shortest genomic island with a length of 5,517 bp. Comparing the average GC content of the genome with 43.68%, it was found that the GC content of the other genomic islands except GIs 006 was lower than the average GC content of the genome. These 15 genomic islands carry a large number of additional functional genes, and the encoded proteins are mainly related to core metabolism, metal ion metabolism, motility chemotaxis, and quorum sensing.

**Table 2:** Prediction results of genomic islands contained in chromosome 1 of FYZ1-3.

Genomic island number	Start position	End position	GI_length (bp)	GC%
GIs 001	115,497	150,760	35,264	42.33
GIs 002	526,592	534,784	8,193	34.16
GIs 003	632,327	642,064	9,738	36.97
GIs 004	1,235,447	1,278,734	43,288	33.98
GIs 005	1,449,628	1,458,581	8,954	38.95
GIs 006	1,674,943	1,683,536	8,594	43.79
GIs 007	1,792,145	1,801,079	8,935	37.59
GIs 008	1,902,091	1,923,017	20,927	38.93
GIs 009	2,081,031	2,096,390	15,360	35.64
GIs 010	2,352,227	2,382,751	30,525	40.45
GIs 011	2,571,091	2,618,109	47,019	36.13
GIs 012	2,793,519	2,808,954	15,436	42.15
GIs 013	3,671,848	3,677,364	5,517	35.49
GIs 014	3,993,864	4,004,575	10,712	33.56
GIs 015	4,132,293	4,142,551	10,259	35.41

#### 3.2.3 Genome difference analysis and collinearity analysis of FYZ1-3 and DSM 10

The chromosome of strain FYZ1-3 was circular with a length of 4,167,567 bp and average GC content of 43.68%. Ori-Finder 2022 was used to annotate the replication origin of the chromosome of the FYZ1-3 strain. The results showed that there were two replication origins in the chromosome. The length of replication origin 1 was 993 nt, the AT content was 0.62, and the location was 4,166,656–4,167,648 bp, which was composed of 15 Dna A boxes. The length of replication origin 2 was 188 nt, the AT content was 0.63, and the location was 1,342–1,529 nt, which was composed of 8 Dna A boxes. From Figure 3B, it can be seen that the chromosome of strain FYZ1-3 has high similarity with most regions of the chromosome of the *B. subtilis* DSM 10 strain and has high homology. However, the chromosome of the FYZ1-3 strain also has some differences; regions 1–4 are unique. After comparison with the predicted genomic islands, regions 1–4 correspond to GIs 004, GIs 008, GIs 011, and GIs 012, respectively.

In general, if the relationship between two species is close, the sequence and order of genes will be very close and is called collinearity (Magain et al., 2017). Collinearity mainly reflects the structural variation between genomes, reflecting the coding sequence and structural homology between genomes. Based on the phylogenetic tree analysis, the model strain *B. subtilis* DSM 10 with the closest homology was selected for Mauve collinearity alignment with the genome of the screening strain FYZ1-3, and the large fragment sequence rearrangement between the genomes was quickly analyzed. It can be seen from Figure 3C that collinearity between strains FYZ1-3 and *B. subtilis* DSM 10 was poor, and there were obvious changes in gene rearrangement time during deletion, inversion, and translocation, indicating that FYZ1-3 strain may evolve a set of phenomena to adapt to the new environmental genome during the high-temperature fermentation of nicotine-containing tobacco waste.

### 3.3 Functional annotation of degrading genes in the functional strain

#### 3.3.1 GO database annotation

**Figure 4:**
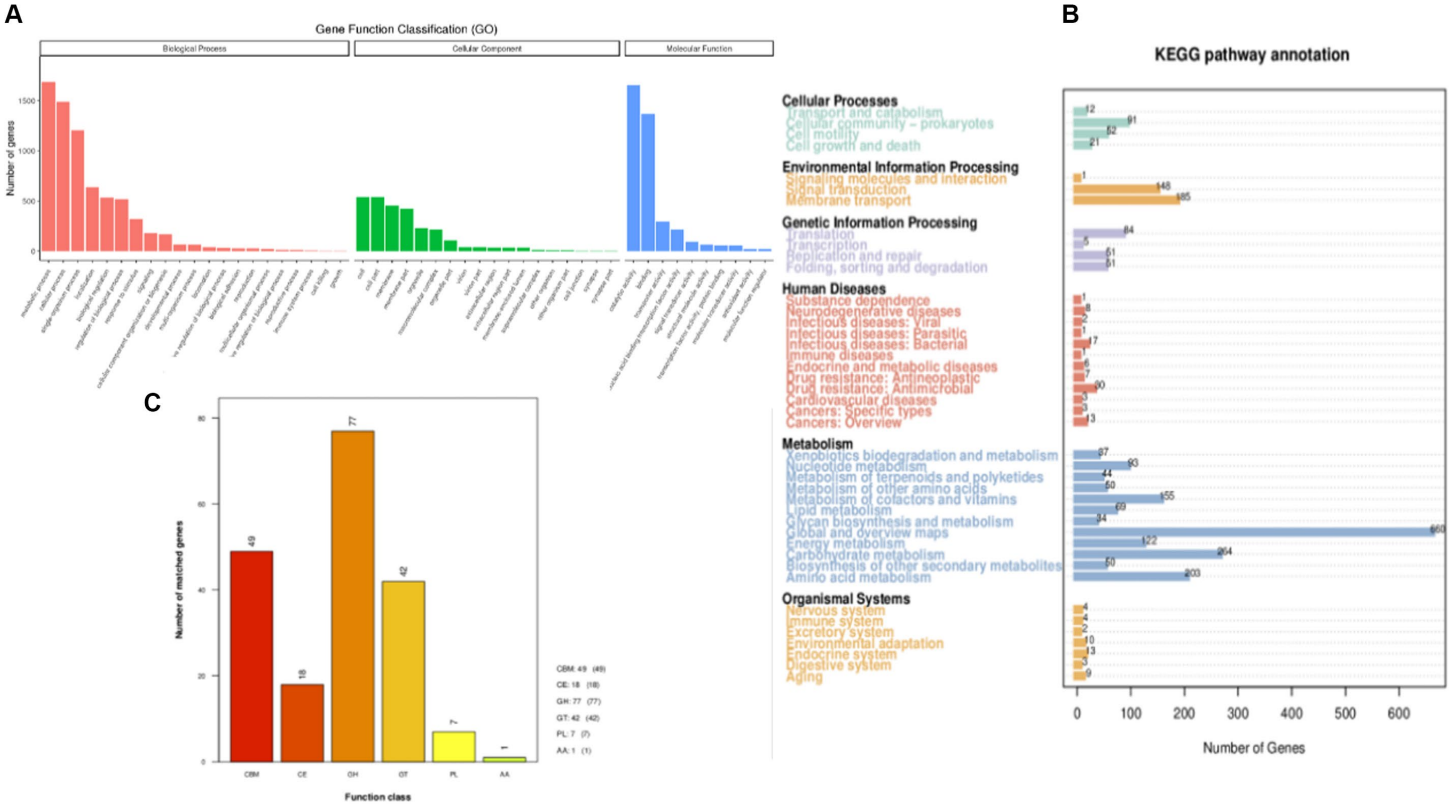
Gene function annotation of the FYZ1-3 strain: **(A)** GO database functional classification; **(B)** KEGG database function classification; **(C)** CAZy database functional classification.

The predicted genes were compared with the GO database using BLAST to obtain the classification annotation information of genes, including cellar components (CC), molecular functions (MF), and biological processes (BP). The statistical results of strain FYZ1-3 in the GO database are shown in Figure 4A. There were 2,926 gene annotations in this classification and a total of 49 functional classifications. According to the MF annotation, 347 genes were involved in FYZ1-3 in the GO-CC classification. The first three functions were cell, cell part, and cell membrane. According to the MF annotation, 1,130 genes were involved in strain FYZ1-3 in the GO-MF classification. The first three functions were catalytic activity, binding, and transport activity, and the gene with amylase activity was *GM000743*. According to the classification of BP, there were 650 GO-BP classifications of genes, and the three most annotated pathways were metabolic processes, cellular processes, and single-cell processes.

#### 3.3.2 KEGG database annotation

KEGG is a database that systematically analyzes the metabolic pathways of gene products in cells and the functions of these gene products. KEGG can be used to further study the complex behavior of genes in the field of biology (Aoki-Kinoshita and Kanehisa, 2007). The genome sequence of strain FYZ1-3 was compared with the KEGG database using BLAST, and the functional annotation results were obtained. A total of 2,502 genes in the KEGG database were functionally annotated on six major functional pathways, namely, cell processes, environmental information processing, genetic information processing, human disease, metabolism, and organism system. The results are shown in Figure 4B. Among them, 1781 genes were annotated in the metabolic pathway. Among the 12 metabolic pathways, 264 genes were related to carbohydrate metabolism, accounting for 14.82% of the annotated genes in the metabolic pathway. Table 3 shows the pathway information of genes related to carbohydrate and protein metabolism. It can be seen that the genome of strain FYZ1-3 contains multiple metabolic pathways and involves a large number of genes, which is one of the reasons why it is efficient in degrading starch and proteins. In addition, 134 genes were annotated at the level of environmental information processing, including 54 genes related to membrane transport and 54 genes related to the two-component signal transduction system. A total of 334 genes were annotated at the level of environmental information processing, including 185 genes related to membrane transport and 148 genes related to the signal transduction system.

**Table 3:** Carbohydrate and protein metabolic degradation pathways and related genes of the FYZ1-3 strain genome.

Item	Metabolic pathway No.	Function description	Gene number
Carbo-hydrate	ko00500	Starch and sucrose metabolism	47
	ko00010	Glycolysis/gluconeogenesis	39
	ko00520	Amino sugar and nucleotide sugar metabolism	43
	ko04973	Carbohydrate digestion and absorption	1
Protein	ko00460	Cyanoamino acid metabolism	5
	ko01230	Biosynthesis of amino acids	122
	ko00250	Alanine, aspartate and glutamate metabolism	33
	ko00471	D-Glutamine and D-glutamate metabolism	6

Furthermore, the KEGG database identified 14 functional genes associated with nicotine metabolism in Table 4. Compared with the genome island information in Table 2, it could be seen that the ppnK gene of FYZ1-3 strain was located on GIs004. Additionally, this strain contained gene clusters namely nadE, nadD, nadA, nadC, and nadB which were related to nicotinamide metabolism, with nadD, nadA, nadC, and nadB located on GIs011 and the pncB gene found on GIs012. From Figure 3B, it was evident that the FYZ1-3 strain possessed three distinctive genomic islands that were absent in the DSM 10 strain. These genes might be responsible for the molecular mechanism of nicotine tolerance in the functional strain.

**Table 4:** Information of functional genes related to nicotine metabolism based on KEGG of the FYZ1-3 strain genome.

Item	Gene_ID	Ko_name	Ko_EC	Identity%	GIs_id
Nicotine	GM000325	nadE	6.3.1.5	100	
	GM000414	gabD	1.2.1.16 1.2.1.79 1.2.1.20	99.6	
	GM000824	yfkN	3.1.4.16 3.1.3.6 3.1.3.5	98.8	
	GM001226	ppnK	2.7.1.23	100	GIs004
	GM001855	pncC	3.5.1.42	99.8	
	GM002193	deoD	2.4.2.1	100	
	GM002298	cca	2.7.7.72 3.1.3.–3.1.4.	100	
	GM002414	punA	2.4.2.1	99.6	
	GM002666	nadD	2.7.7.18	99	GIs011
	GM002885	nadA	2.5.1.72	99.7	
	GM002886	nadC	2.4.2.19	99.7	
	GM002887	nadB	1.4.3.16	99.2	
	GM003111	ppnK	2.7.1.23	100	GIs012
	GM003348	pncB	6.3.4.21	99.8	

#### 3.3.3 CAZy database annotation

The CAZy database includes carbohydrate-related enzyme families that catalyze carbohydrate degradation, modification, and biosynthesis. The enzyme family is divided into the following six categories: glycoside hydrolase (GH), polysaccharide lyase, carbohydrate esterase, glycosyltransferase, auxiliary activity (AA), and carbohydrate-binding module (CBM). The annotation results are shown in Figure 4C. There are 194 CAZy enzyme gene families encoded by the FYZ1-3 strain, of which the GH family accounts for the highest (39.7%) of the whole gene family, followed by the CBM family, accounting for approximately 25.3%. GH, as a key component of carbohydrate metabolism, can catalyze the hydrolysis of glycosidic bonds in various glycosyl compounds. CBM is a noncatalytic protein domain that binds to carbohydrates. It is a polysaccharide-binding module that does not have catalytic activity but participates in carbohydrate degradation. It can specifically bind to polysaccharides and effectively improve the catalytic efficiency of carbohydrate-degrading enzymes. The main components of tobacco waste are carbohydrates and proteins, and the FYZ1-3 strain contains genes encoding endoglucanase (lichenase/endo-beta-1,3-1,4-glucanase [EC 3.2.1.73], β-glucosidase [β-glucosidase, EC 3.2.1.21], α-amylase [amyE, EC 3.2.1.1], and other enzymes). From the perspective of gene analysis, the degradation mechanism of the FYZ1-3 strain on tobacco waste was further understood.

### 3.4 Biological control effect of the functional strain

#### 3.4.1 Prediction of the secondary metabolic gene cluster of the FYZ1-3 strain

**Figure 5:**
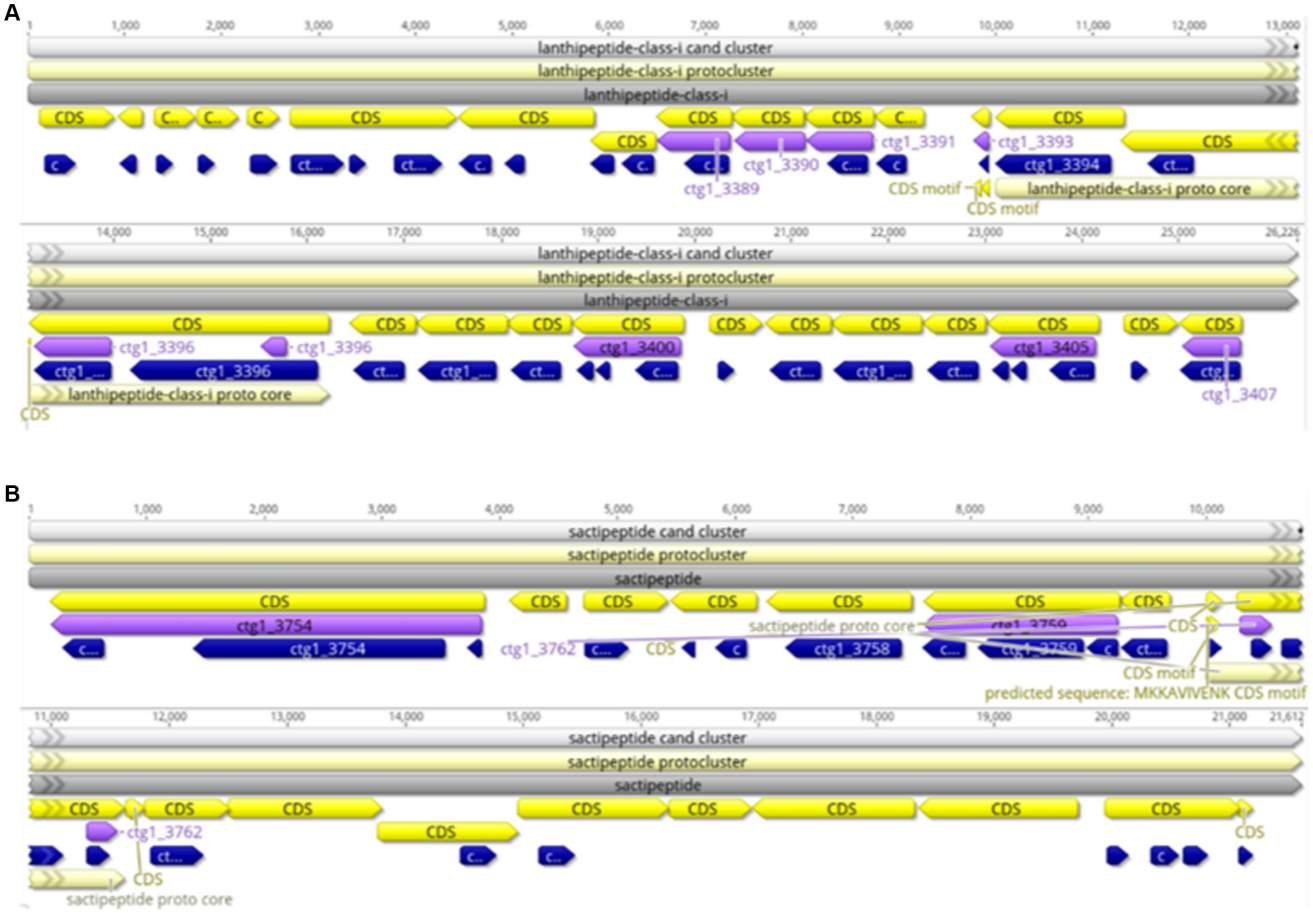
Linear map of the wool thiopeptide antibiotic synthesis gene cluster of strain FYZ1-3: **(A)** cluster 8 lanthipeptide-class-i; **(B)** cluster 9 sactipeptide.

AntiSMASH was used to predict and analyze the secondary metabolite synthesis gene cluster of FYZ1-3. There were 10 secondary metabolites in total. The specific prediction analysis results are shown in Table 5. After comparing all gene clusters of strain FYZ1-3 with the known secondary metabolite gene clusters using BLAST, 8 functional synthetic gene clusters and 2 unknown synthetic gene clusters were found, which indicated the likelihood of new active substance synthesis gene clusters in the gene sequence of FYZ1-3. Lignosulfan is a heat-stable antimicrobial peptide on the cell membranes of Gram-positive bacteria, which can be used as a substitute for antibacterial drugs (Willey and Donk, 2007). Similarity between the Willey J., Donk wool sulfur antimicrobial peptide gene cluster 8 and lanthipeptides: Bateq7PJ16_RS18530 in the genome of *B. subtilis* FYZ1-3 reached 100%, and the similarity between gene cluster 9 and sactipeptides: BSU_37350 reached 100%. Thus, it may be used as a development strain for the synthesis of wool sulfur antibiotics (Figure 5). The strain contains 44 wool sulfur antimicrobial peptide gene clusters, which are related to the antibacterial properties of bacilli. The annotated sactipeptide is a class of polypeptides with an intramolecular thioether bond connected by a cysteine sulfhydryl group and an α-carbon atom of the receptor amino group. It is formed by free radical S-adenosylmethionine enzyme-dependent catalysis of the propeptide. This unique bond is completely different from the bond that connects sulfur-containing amino acid residues to β-carbons (Flühe and Marahiel, 2013).

**Table 5:** Prediction and analysis of the secondary metabolite gene cluster of FYZ1-3.

Gene cluster number	Gene dosage	Gene cluster type	The most similar gene cluster	Similarity/%
Cluster 1	26	NRPS	Surfactin	82
Cluster 2	20	Terpene	—	—
Cluster 3	55	NRPS, PKS-like, T3PKS, transAT-PKS	Bacillaene	100
Cluster 4	37	NRPS, betalactone	Fengycin	100
Cluster 5	18	Terpene	—	—
Cluster 6	44	T3PKS	1-carbapen-2-em-3-carboxylic-acid	16
Cluster 7	44	NRP-metallophore, NRPS	Bacillibactin	100
Cluster 8	26	Lanthipeptide-class-i	Subtilin	100
Cluster 9	18	Sactipeptide	Subtilosin A	100
Cluster 10	39	Other	Bacilysin	100

Bacteriocin is a polypeptide with antibacterial activity synthesized by bacteria during the metabolic process. It is a naturally found microbial preservative that is safe and nontoxic with strong antibacterial activity and mild action conditions (Holo et al., 1991). According to the results from previous studies, the antibacterial substances produced by *Bacillus* are mainly lipopeptide antibiotics and polyketide antibiotics, which have antagonistic activity against several pathogenic bacteria. The application of these substances to soil can enhance plants’ own resistance (Ongena and Jacques, 2007). Based on the prediction of the secondary metabolite gene cluster, it was found that the genome of strain FYZ1-3 contained multiple genes related to bacteriocins, and it was speculated that it had certain antibacterial properties.

#### 3.4.2 Analysis of antibacterial effect gene of the FYZ1-3 strain

**Figure 6:**
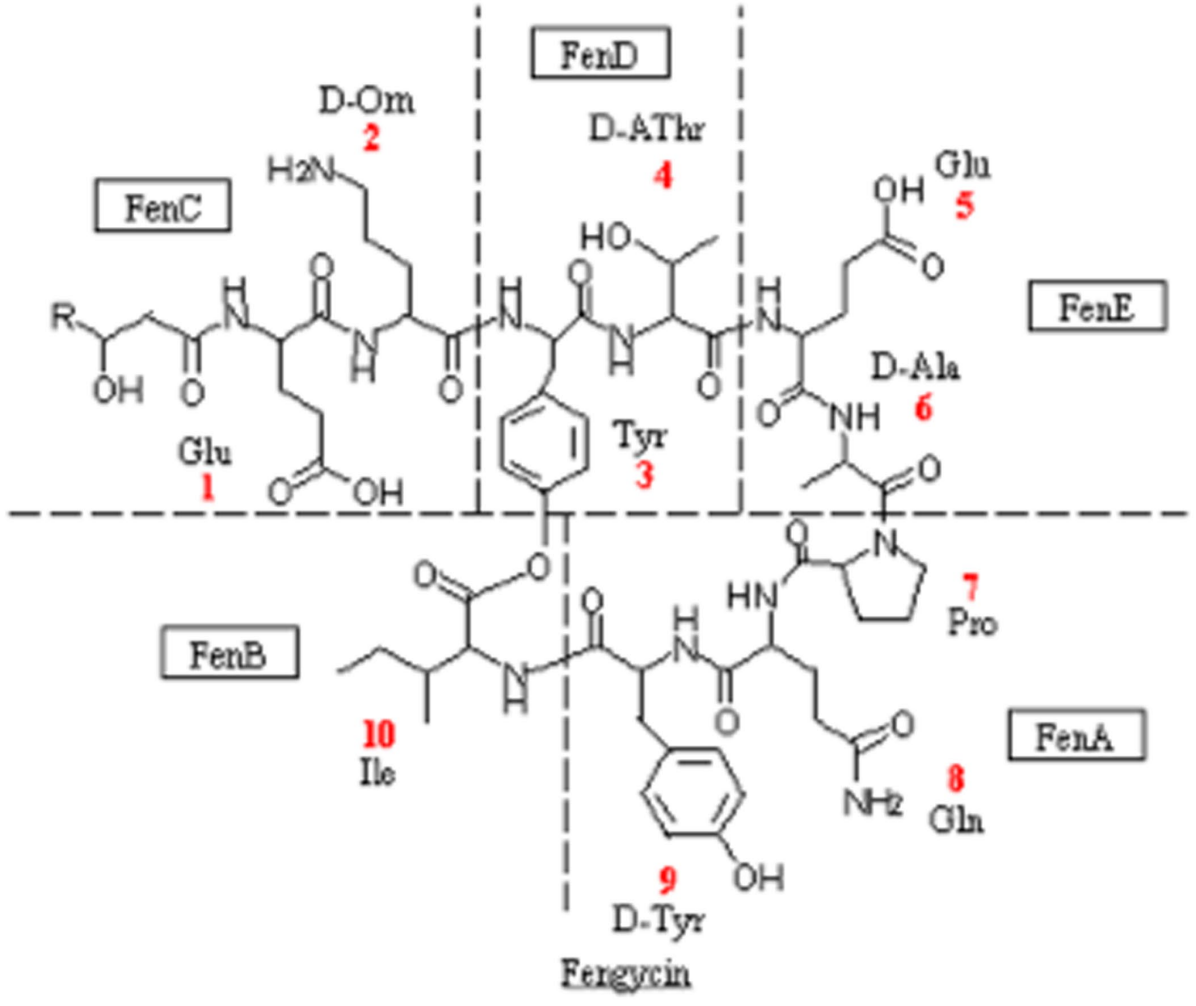
Structural representation and synthetic gene of fengycin.

Fengycin and bacilysin are the most common antibacterial substances in *Bacillus*, which can be synthesized by non-ribosomal pathway with a molecular weight ranging from 300 to 3,000 μ. Existing studies have demonstrated the effectiveness of lipopeptide antimicrobial compounds, including surfactin, iturin, and fengycin, which are produced by the lipopeptide synthase gene cluster, against various plant pathogenic bacteria, fungi, and oomycetes (Meena and Kanwar, 2015; Abdalah et al., 2018). Among them, fengycin synthase genes had higher antibacterial activity (Li et al., 2012; Chen et al., 2019). According to the KEGG database annotation, fengycin and bacilysin synthase regulatory genes could be detected in FYZ1-3 strain, including 5 genes of fengycin and 7 genes of bacilysin, as shown in Table 6. The molecular structure of fengycin contains a circular part composed of 10 amino acids and a long-chain fatty acid branch chain. The synthetic gene is composed of five genes: *fenC, fenD, fenE, fenA* and *fenB*, as shown in Figure 6. FYZ1-3 strain had complete Fengycin synthase regulatory genes, indicating a strong inhibitory effect on filamentous fungi. Based on the prediction of secondary metabolite gene clusters and KEGG antibacterial genes, it was found that the genome of strain FYZ1-3 contained multiple bacteriocin-related gene clusters, indicating that it could possess antibacterial qualities.

**Table 6:** Information of bacteriostasis genes based on KEGG of FYZ1-3.

Type	Gene _ID	Identity%	Ko_id	Ko_name	Function
Fengycin	GM002040	99.1	K15668	ppsE, fenB	Fengycin family lipopeptide synthetase E
	GM002041	97.7	K15667	ppsD, fenA	Fengycin family lipopeptide synthetase D
	GM002042	98.5	K15666	ppsC, fenE	Fengycin family lipopeptide synthetase C
	GM002043	98.9	K15665	ppsB, fenD	Fengycin family lipopeptide synthetase B
	GM002044	98.5	K15664	ppsA, fenC	Fengycin family lipopeptide synthetase A
Bacilysin	GM003982	99.6	K19550	bacG	Bacilysin biosynthesis oxidoreductase BacG
	GM003983	99.7	K19549	bacF	Bacilysin biosynthesis transaminase BacF
	GM003984	100	K19552	bacE	MFS transporter, DHA3 family, bacilysin exporter BacE
	GM003985	99.8	K13037	bacD	L-alanine-L-anticapsin ligase Dihydroanticapsin dehydrogenase 3-[(4R)-4-hydroxycyclohexa-1,5-dien-1-yl]-2-oxopropanoate isomerase Prephenate decarboxylase
	GM003986	98.8	K19548	bacC	
	GM003987	99.6	K19547	bacB	
	GM003988	100	K19546	bacA	

#### 3.4.3 Verification of the fungal-inhibition ability of the FYZ1-3 strain

**Figure 7:**
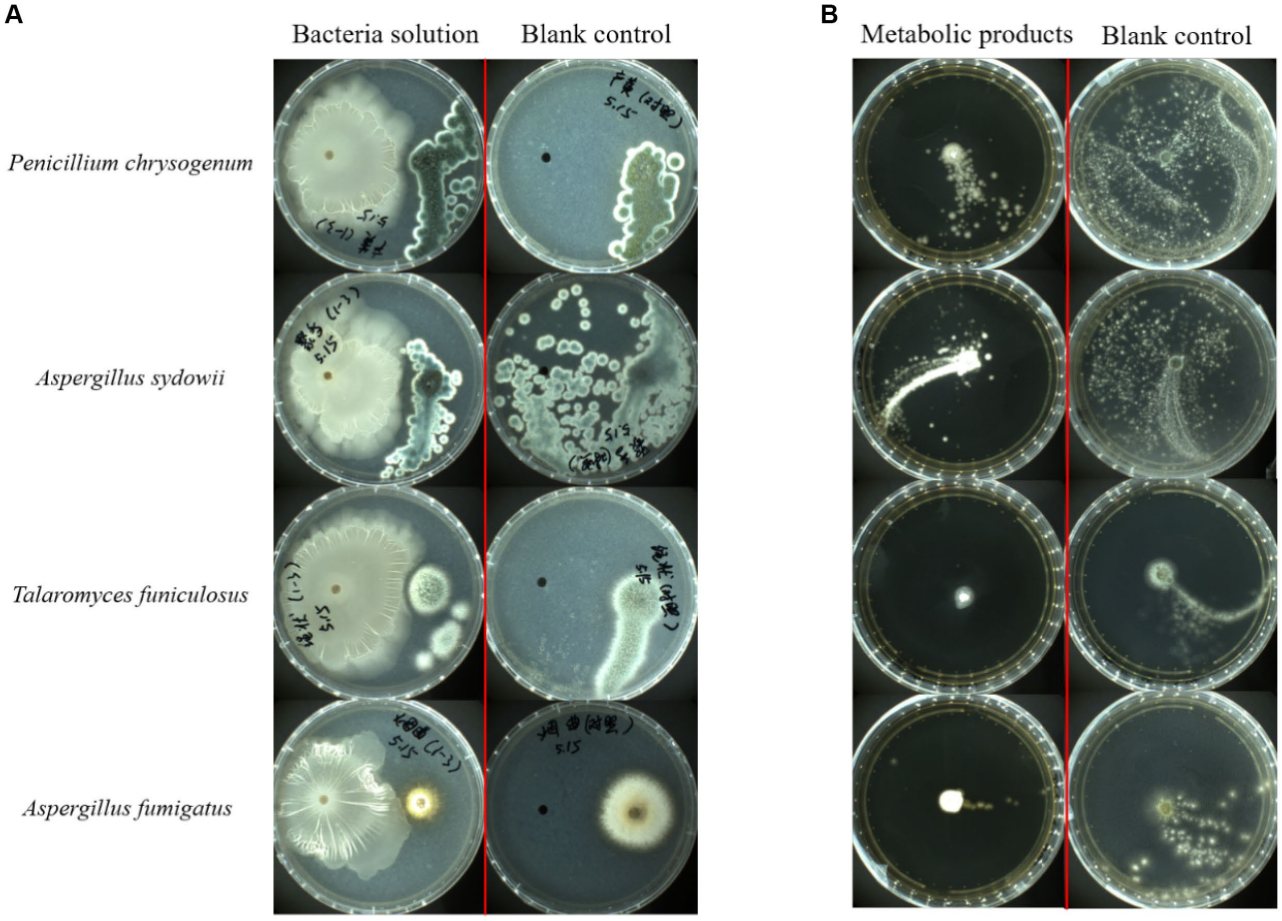
Bacterial inhibition by strain FYZ1-3: **(A)** growth results using FYZ1-3 bacterial solution in the plate confrontation method for 72 h; **(B)** 48 h growth results of FYZ1-3 in the metabolite agar column method.

Four common pathogenic fungi in tobacco waste, *P. chrysogenum*, *A. sydowii*, *A. fumigatus*, and *T. funiculosus* were selected for plate confrontation experiments to determine the inhibitory ability of FYZ1-3 to fungi. The growth of the strain after 72 h of culture at 28°C is shown in Figure 7A. No filamentous fungal growth was observed around strain FYZ1-3, and the colony surface in the *P. chrysogenum* group could not form lemon-yellow droplets by normal exudate accumulation. The spores of *A. sydowii* in the experimental group could not be dispersed in the whole culture dish similar to that in the control group. Although multiple colonies were formed in the experimental group of *T. funiculosus*, no sterile colonies were growing around strain FYZ1-3. The *A. fumigatus* colony in the experimental group was small, and exhibited obviously shrunken mycelia. In summary, strain FYZ1-3 had an obvious growth-inhibition effect on the four fungi.

Since there were two microorganisms grown on a culture medium in the antagonistic experiment, there was a competition for limited space and nutrients. The observed inhibitory effect might be due to the low growth competition rate of the fungi rather than the secretion of growth inhibitors by the strain FYZ1-3, as the functional bacteria had a growth rate significantly exceeding the fungi. To eliminate this confounding factor, the agar column method was employed, which inoculated a single colony of filamentous fungi onto PDA culture medium containing sterile fermentation supernatant from functional bacteria FYZ1-3. The growth state of fungi was then observed in the absence of other microorganisms. In this way, the growth of fungi was dependent solely on the metabolic products produced by the added functional microorganisms.

The growth of fungal strains was recorded after 48 h of culture at 28°C (Figure 7B). The mycelium of *P. chrysogenum* and *A. sydowii* in the control group had spread throughout the culture dish, whereas fungal growth in the culture dish containing the fermentation supernatant of FYZ1-3 strain was strongly inhibited by up to 75.8%. Observation of the colonies of *T. funiculosus* and *A. fumigatus* revealed only a small amount of diffusion at the edge of the inoculated fungal colony in the experimental group, and a small amount of mycelium was found growing in other parts of the culture dish. The color and morphology were significantly different from those of the normally growing mycelium in the control group. There was some *Penicillium* growth in the Petri dish to which the sterile fermentation supernatant of the FYZ1-3 strain was added. The inhibition rates of the four groups were calculated to be 75.8, 63.4, 48.6, and 44.1%, respectively. Observation of the colony morphology revealed that the experimental group had larger fungal colonies with more loose hyphae and unclear boundaries. The control group had smaller colonies, dense hyphae, and clear boundaries.

Based on these experiments, it could be inferred that the fermentation supernatant of the FYZ1-3 strain contains substances that can inhibit the growth and development of fungi, inhibit the germination of spores and bacterial growth, and change the normal morphology of mycelium. Therefore, strain FYZ1-3 is the target strain that can inhibit fungal growth.

## 4 Discussion

Currently, *Bacillus* spp., as the most commonly selected representative, has become the core genus of effective microorganisms (EM) and other commercial bacterial agents. *Bacillus* spp. can produce several extracellular enzymes such as amylase, protease, and cellulase, while its thicker cell wall and spores can help the bacterium survive in harsh high-temperature and high-salt environments (Sonenshein et al., 1993). There are many studies on the degradation mechanism and applications of *B. subtilis*; however, owing to the unique structure and composition of organic substances in tobacco sources, as well as the presence of the biological toxin nicotine in tobacco waste, bacterial agents that have been identified and demonstrated to show potential still cannot be directly used for the aerobic degradation of tobacco waste. Therefore, the search for functional strains that are resistant to nicotine and have a high degradation ability for tobacco source organic matter has important scientific significance and practical value in improving the resource-utilization rate of tobacco waste.

Understanding the basic genomic information of functional strains allows researchers to further understand the mechanism of metabolic transformation of functional strains and facilitates understanding the relationship between genes and proteins, metabolic functions, and individual behaviors. Therefore, genomics plays a crucial role in understanding the physiological performance and ecological significance of functional bacteria (Seminago et al., 2022). In this study, the genome of *B. subtilis* FYZ1-3, a strain isolated from tobacco waste compost, was analyzed. Based on phylogenetic analysis and metabolic pathway analysis of its genome, and comparative analysis with the genome of the standard strain DSM 10, the evolutionary development status and metabolic potential of the functional strain FYZ1-3 in *B. subtilis* were revealed at the molecular level. Moreover, the mobile genome islands carrying a large number of functional genes were identified, providing an important basis for gene function analysis and environmental niche research.

Due to the wide antibacterial spectrum and strong adaptability, *B. subtilis* has been increasingly used in the treatment of diseases in humans, animals, and plants, as well as biosurfactants, food preservatives, feed additives, and molecular biology research tools. Its antibacterial mechanism was mainly manifested in competition, antagonism, and induction of biological resistance. This study aimed to improve the efficiency of aerobic fermentation degradation of tobacco waste and produce high-quality organic fertilizers. A strain of *B. subtilis* FYZ1-3 was screened, and its biological control ability against common pathogenic fungi was speculated through genome analysis and laboratory experimentation. Therefore, this functional strain FYZ1-3 presented itself as a crucial contender for microbial organic fertilizers, providing a harmonious blend of organic fertilizers and advantageous microorganisms. Organic fertilizers could create an optimal environment for microorganisms, extending their survival and enhancing their ability to control soil pathogens. The implementation of microbial organic fertilizers could increase the treatment of tobacco waste, minimize secondary pollution, and promote green circular development. Moreover, it could curtail the incidence of soil-borne diseases by suppressing the activity of pathogenic microorganisms in the soil due to the antibacterial impact of FYZ1-3.

## 5 Conclusion

This study isolated a functional strain, FYZ1-3, from aerobic fermentation samples of tobacco waste, which could tolerate high temperatures up to 80°C and nicotine concentrations as high as 0.6%. FYZ1-3 exhibited high degradation ability towards tobacco starch and tobacco protein, with amylase and protease activities of 122.3 U/mL and 52.3 U/mL, respectively. Through morphology and molecular biology analysis, FYZ1-3 was identified as *B. subtilis*. Using whole genome sequencing, the reasons for high carbohydrate and protein degradation rates and nicotine tolerance were analyzed at the molecular level, and the ability of FYZ1-3 strain to inhibit fungal growth was identified and validated. This study marks the initial step in exploring and studying the functional strain FYZ1-3 for tobacco waste treatment. In the future, we will continue to explore and verify the functional genes through molecular biology, and conduct material and genetic analysis of metabolites. To summarize, FYZ1-3 exhibits promising potential as a microbial preparation candidate for utilizing tobacco waste resources, based on our research findings. This study enhances microbial resources and establishes a theoretical basis for enhancing aerobic fermentation efficiency and product utilization for tobacco waste.

## Data availability statement

The datasets presented in this study can be found in online repositories. The names of the repository/repositories and accession number(s) can be found in the article/supplementary material.

## Author contributions

CY: Conceptualization, Methodology, Formal analysis, Funding acquisition. DaL: Investigation, Validation, Methodology, Formal analysis, Data curation. KH: Methodology, Visualization. DoL: Resources, Methodology, Project administration. XM: Data curation, Visualization. YJ: Investigation, Methodology. HX: Methodology.
